# Detection of lymph node metastasis in non-small cell lung cancer using the new system of one-step nucleic acid amplification assay

**DOI:** 10.1371/journal.pone.0265603

**Published:** 2022-03-21

**Authors:** Naoko Ose, Yukiyasu Takeuchi, Yasushi Sakamaki, Yoshihisa Kadota, Koji Urasaki, Hiromi Tsuji, Kunimitsu Kawahara, Mayuko Noguchi, Yasushi Shintani

**Affiliations:** 1 Department of General Thoracic Surgery, Osaka University Graduate School of Medicine, Osaka, Japan; 2 Department of General Thoracic Surgery, Osaka Toneyama Medical Center, Osaka, Japan; 3 Department of Chest Surgery, Osaka Police Hospital, Osaka, Japan; 4 Department of General Thoracic Surgery, Osaka Habikino Medical Center, Osaka, Japan; 5 Department of Laboratory Medicine and Pathology, Osaka Toneyama Medical Center, Osaka, Japan; 6 Department of Pathology, Osaka Police Hospital, Osaka, Japan; 7 Department of Pathology, Osaka Habikino Medical Center, Osaka, Japan; 8 Gene Testing Business, Sysmex Corporation, Hyogo, Japan; Osaka Medical Center for Cancer and Cardiovascular Diseases, JAPAN

## Abstract

**Introduction:**

The prognosis of non-small cell lung cancer greatly depends on the presence of lymph node metastasis, which limits the need for surgery and adjuvant therapy for advanced cancer. One-step nucleic acid amplification of cytokeratin19 (CK19) mRNA was used to detect lymph node metastasis. Automated Gene Amplification Detector RD-200 and the LYNOAMP CK19 gene amplification reagent as components of the new one-step nucleic acid amplification system, which has increased gene amplification efficiency by improving the reagent composition, have shorter preprocessing and measurement times than conventional systems. We aimed to compare the clinical performance of the new system with that of histopathology and the conventional system.

**Materials and methods:**

199 lymph nodes from 58 non-small cell lung cancer patients who underwent lymph node dissection were examined intraoperatively using the new system, conventional system, and histopathology.

**Results:**

Lymph node metastasis was diagnosed in 32, 42, and 44 patients using histopathological analysis, the new system, and the conventional system, respectively. Compared with histopathological analysis, the concordance rate, sensitivity, specificity, positive predictive value, and negative predictive value of the new system were 92.0%, 90.6%, 92.2%, 69.0%, and 98.1%, respectively, and compared with the conventional system, the values were 95.0%, 86.4%, 97.4%, 90.5%, and 96.2%, respectively.

**Conclusion:**

The clinical performance of the new one-step nucleic acid amplification system in detecting lymph node metastasis of lung cancer is comparable to that of histopathology and the conventional system; its performance was sufficient for determining the appropriate clinical treatment. The new rapid system can be effectively utilized during lung cancer treatment intraoperatively and postoperatively.

## Introduction

The incidence of lung cancer is increasing each year, with 2.09 million new cases and 1.76 million deaths worldwide reported in 2018, both of which are the largest in the category worldwide [1]. The prognosis of non-small cell lung cancer (NSCLC), which accounts for 85% of all lung cancer cases, greatly depends on the presence of lymph node metastasis [2]. Surgical treatment is performed for patients with disease stages 0, I, and II who developed metastasis only in the hilar lymph node, and lobectomy and systematic lymphadenectomy are the standard operative methods [3]. If the primary tumor measures 2 cm or less in diameter and no lymph node metastasis is confirmed, limited surgeries, such as segmentectomy, may be selected instead of lobectomy to preserve the pulmonary function [4]. In patients without lymph node metastasis, selective lymph node dissection is considered to reduce the operation time and patient burden [5]. Intraoperative diagnosis of lymph node metastasis may prompt a change in operative methods. Postoperative diagnosis of lymph node metastasis may prompt a change in adjuvant therapy for advanced cancer.

Thus, an accurate intra- and postoperative diagnosis of lymph node metastasis is required. Although lymph node metastasis is usually diagnosed histopathologically using resected specimens, one-step nucleic acid amplification (OSNA), a genetic test, is also effective for the diagnosis of lymph node metastasis. The OSNA assay, a new method developed in 2007, measures the tumor cell-specific expression of cytokeratin 19 (CK19) mRNA in lymph nodes and determines the presence of lymph node metastasis by comparing the level to a cut-off value. Inoue et al. reported that OSNA assay measuring CK19 was more useful than using other target markers in lung cancer [6]. OSNA has shown clinical performance equivalent to that of the gold standard technique of performing histopathology using a formalin-fixed paraffin-embedded slide for the diagnosis of regional lymph node metastasis in patients with breast, colorectal, and gastric cancer as well as NSCLC [7–18]. Owing to its simplicity, accuracy, and rapidity, the guidelines recommended intraoperative sentinel lymph node biopsy, especially for breast cancer [19–25]; this method would be useful for the diagnosis of lymph node metastasis in patients treated for lung cancer.

Although RD-100i and its reagent LYNOAMP BC have been primarily used for the OSNA method, the RD-200 and LYNOAMP CK19 systems have been developed as an improved new system [26]. An increase in the efficiency of gene amplification by improving the reagent composition led to shorter preprocessing and measurement times than the conventional systems; in two lymph nodes, a reduction of 9 min from lymph node solubilization to diagnosis was achieved. Improvements have also increased the number of specimens (from 4 to 14) that can be measured simultaneously, making it easier to use for postoperative diagnosis, thus relatively measuring a large number of lymph nodes. The efficiency of the new system is comparable to that of the conventional system in terms of diagnosing breast [14], colorectal, and gastric cancers. If the new system is effective for the diagnosis of lymph node metastasis in patients with NSCLC, it would also be beneftial in patients treated for lung cancer. Therefore, we compared the clinical performance of the new system with that of histopathology and the conventional system and aimed to demonstrate that the new system has the same or better diagnostic capability. In addition, the effect of contamination of the lung parenchyma, some of which have high CK19 expression, and the ability to search for metastases on small lymph nodes examined using the new system.

## Materials and methods

### Patients

A total of 58 patients with NSCLC who underwent surgical resection at Osaka University Hospital, Osaka Toneyama Medical Center, Osaka Police Hospital, and Osaka Habikino Medical Center between December 2018 and November 2019 were enrolled in the study. A total of 199 lymph nodes were included. Patients who had active double cancers, were treated with neoadjuvant therapy, had another organ cancer within 5 years, or were deemed incapable of participating in the study by an investigator were excluded. Lymph node specimens were randomly obtained from patients with a minor axis of 5 mm or more, and fewer than five specimens were obtained from each patient. All patients were informed of the study and provided a written consent. This study was initially approved by the Institutional Review Board of Osaka University Hospital (approval no. 18248) in November 2018 and thereafter by each participating institution.

#### Sample size setting for correlation proof

The number of samples and the criteria of clinical performance required for correlation should be such that "the concordance rate is at least 90% when the results are statistically processed using a cohort including at least 50 positive and negative samples". That is the concordance rate of 90% using at least 100 samples was the minimum requirement. In this case, the 95% confidence interval for the concordance rate was calculated to range from 83% to 95%. Therefore, the authors considered the threshold of the concordance rate to be 83% to indicate equivalence. Based on the results of the feasibility study comparing with the conventional and new systems, the expected concordance rate in this study was 92%. In this case, the number of samples required was calculated to be 146 to ensure that the lower limit of the 95% confidence interval was not less than the threshold value of 83% at a significance level (α) of 5% and a statistical power (1-β) of 90%.

The frequency of histological types other than adenocarcinoma and squamous cell carcinoma in non-small cell lung cancer cases is approximately 20%. We wanted to include approximately 10 cases of histological types other than adenocarcinoma and squamous cell carcinoma in our cohort to evaluate their effects. Therefore, based on these frequencies, the number of cases required for this study was at least 50. Finally, the number of samples required in this study was set at a minimum of 200, assuming that an average of four lymph nodes would be collected per case.

### Methods

The metastasis detection accuracy of RD-200/LYNOAMP CK19 (the new system) was compared with that of histopathology and RD-100i/LYNOAMP BC (the conventional system).

#### Lymph node processing for OSNA and histopathology

**Fig 1:**
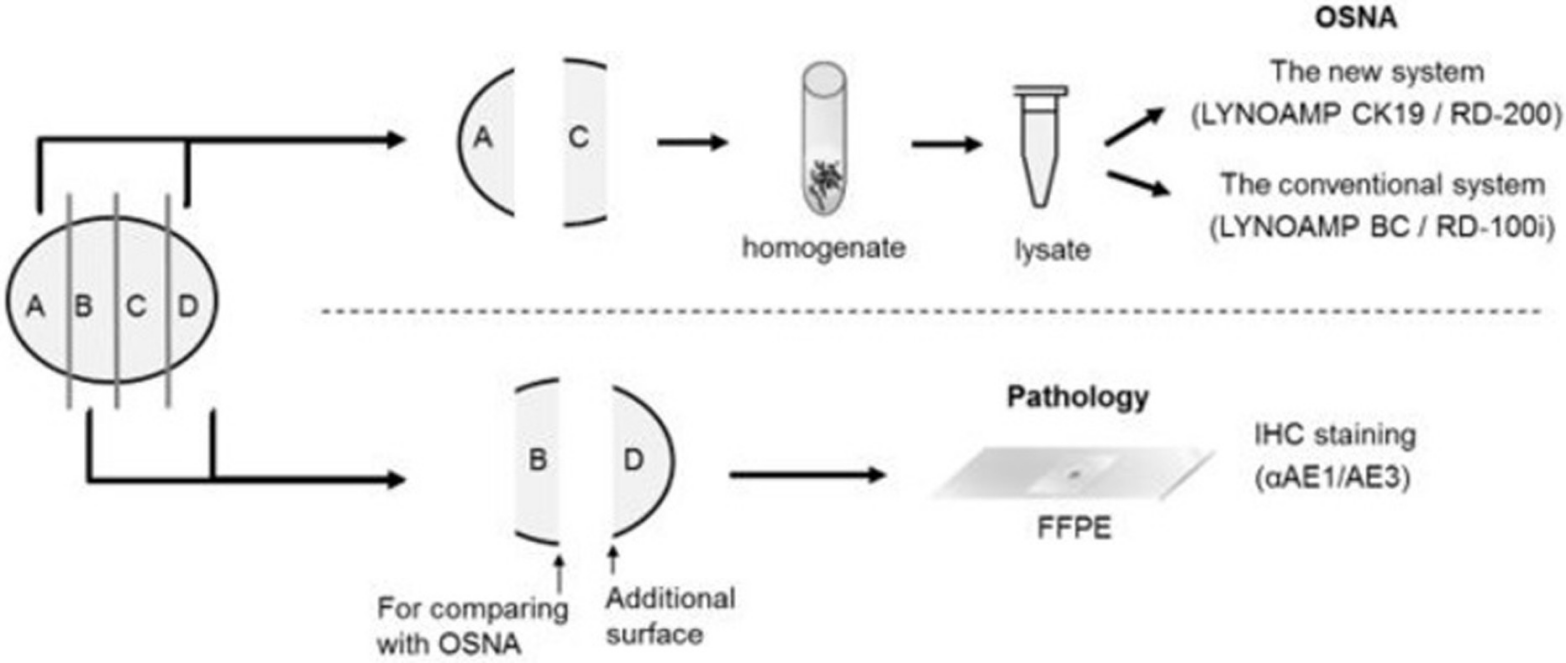
Schematic representation of lymph node preparation. The LN specimens were cut into four pieces. Blocks “A” and “C” were subjected to the OSNA assay, and blocks “B” and “D” were subjected to histopathological analysis with hematoxylin and eosin staining and an IHC assay. OSNA, one-step nucleic acid amplification; IHC, immunohistochemical; FFPE, formalin-fixed paraffin-embedded; AE1/AE3, pan-cytokeratin.

The minor axis was measured using a ruler. The lymph nodes were cut into four blocks (Fig 1), two of which (blocks A and C) were quickly frozen for the OSNA assay, while the other blocks (blocks B and D) were embedded in paraffin and subjected to histopathology.

#### Histopathology

The surfaces of blocks B and D were subjected to histopathological analysis. The lymph nodes serving as tissue slices were embedded in paraffin and subjected to histopathology via immunohistochemistry using a monoclonal mouse anti-human keratin/cytokeratin (AE1/AE3) antibody (Nichirei Bioscience Inc., Tokyo, Japan) to detect lymph node metastasis and via hematoxylin and eosin staining to detect the contamination of lung epithelial cells. The histopathological results of both block B and block D were used to identify discordant cases in order to evaluate the new OSNA system and provide a comparison between this system and the traditional histopathological method performed for patients with NSCLC, which can only diagnose the median single surface. To compare the size of lymph nodes and accurately assess the presence of metastasis in the small lymph nodes, the lymph node was examined and diagnosed as positive if either or both blocks B and D were diagnosed as positive. Lymph node metastasis was classified by histopathology based on the Cancer Staging Manual of the American Joint Committee on Cancers, 8^th^ edition [27] (Table 1).

**Table 1:** Diagnostic characteristics of histopathology, conventional system, and new system.

Pathological diagnosis gold standard		OSNA	Conventional system^a^	New system^b^
Positive	Macrometastasis: The metastatic lesions in the greatest dimension measure >2 mm.	Positive	[CK19 mRNA] ≥250 copies/μL	[CK19 mRNA] ≥250 cCP/μL
	Micrometastasis: The metastatic lesions in the greatest dimension measure ≤2 mm and >0.2 mm.			
Negative	Isolated tumor cells (ITCs): The metastatic lesions in the greatest dimension measure ≤0.2 mm or <200 metastasis-positive cells.	Negative	[CK19 mRNA] <250 copies/μL	[CK19 mRNA] <250 cCP/μL
	No metastasis: No metastasis-positive cells were found.			

a: The conventional system shows “I” flag for positive specimens, which strongly interferes with gene amplification.

b: cCP is the unit that indicates conversion from the new system to the conventional one.

#### Analysis of lymph nodes using the conventional and new system

Lymph node blocks A and C were homogenized in 4 mL of lysis buffer LYNORHAG (Sysmex Corporation, Hyogo, Japan) with RP-10 (Sysmex Corporation, Hyogo, Japan) and a LYNOPREP blade set (Sysmex Corporation, Hyogo, Japan). Each lymph node homogenate was centrifuged at 10,000 × *g* for 60 s at room temperature. The intermediate aqueous layer was regarded as the lymph node lysate. It was diluted 10-fold with LYNORHAG in a sample container on ice (0°C–4°C) and mixed by vortexing. This solution was considered as the analysis sample. The analysis sample was further diluted with LYNORHAG in a sample container on ice (0°C–4°C) and mixed by vortexing. This solution was considered as the diluted sample. Both the analysis samples and diluted samples were analyzed using the conventional system, whereas only the analysis samples were analyzed using the new system following the manufacturer’s instructions. The conventional system and new system automatically identified each sample as positive or negative according to the criteria listed in Table 1. The new system is more resistant to inhibition reactions by specimen-derived components than the conventional system. Therefore, the CK19 level (copies/μL) in the sample was higher than that in the conventional system. The new system automatically reports the value converted as the equivalent value of the conventional system as “cCP.” The OSNA assay was performed three times to evaluate the discordance between the two methods.

#### Statistical analysis

The variables were expressed as mean ± standard deviation. All analyses were performed using the JMP 15.1.0 statistical software package (SAS Institute Inc., USA).

## Results

### Study participants

A total of 199 lymph nodes resected from 58 patients from four institutions were included in this study. The age and clinical characteristics of the patients are shown in Table 2. Three patients underwent sampling of mediastinal lymph node only because of dissemination in two patients who were diagnosed with pathological stage IVa and in one patient who was diagnosed multiple mediastinal lymph node metastasis.

**Table 2:** Patients’ characteristics.

Characteristics		Characteristics	
Age (years)		Dissected lymph nodes	
Median	70	ND1b	3
Range	36–87	ND2a-1	19
Patients		ND2a-2	32
Male	43	ND2b	1
Female	15	Lymph node sampling	3
Tumor histology		Pathological stage	
Adenocarcinoma	40	IA	10
Squamous cell carcinoma	14	IB	9
Adenosquamous cell carcrcinoma	1	IIA	6
Pleomorphic carcinoma	1	IIB	14
Large cell carcinoma	1	IIIA	16
Large cell neuroendcrine carcinoma	1	IIIB	1
Pathological N status		Unknown*	2
N0	31	Mode of resection	
N1	15	Pneumonectomy	1
N2	9	Lobectomy	53
N3	1	Sleeve resection	1
Unclassified	2	Lymph node sampling	3

*Unclassified: Only lymph node sampling was performed, and pathological N staging was unclassified.

### Diagnostic performance of the new system, histopathology, and conventional system

Table 3 shows the comparison of diagnostic performance among the new system, histopathology, and conventional system using lymph node specimens. Compared with histopathology, the concordance rate, sensitivity, specificity, positive predictive value (PPV), and negative predictive value (NPV) of the new system were 92.0% (95% CI: 87.4%–95.0%), 90.6% (95% CI: 75.8%–96.7%), 92.2% (95% CI: 87.5%–95.4%), 69.0% (95% CI: 53.5%–80.9%), and 98.1% (95% CI: 94.5%–99.3%), respectively. The discordant cases determined through histological examination consisted of 13 false-positive and 3 false-negative specimens. The concordance rate, sensitivity, and specificity of the new system relative to histopathology were equivalent to those of the conventional system, as shown in the current reports [7, 8]. Compared with the conventional system, the concordance rate, sensitivity, specificity, PPV, and NPV of the new system were 95.0% (95% CI: 91.0%–97.3%), 86.4% (95% CI: 73.3%–93.6%), 97.4% (95% CI: 93.5%–99.0%), 90.5% (95% CI: 78.2%–96.2%), and 96.2% (95% CI: 91.9%–98.2%), respectively. Therefore, it was confirmed that the performance of the new system was equivalent to that of the conventional system.

**Table 3:** Comparison of diagnostic performance between the new system and histopathology or the conventional system.

New system	Histology		Conventional system		Total
	Positive	Negative	Positive	Negative	
Positive	29 (14.6%*)	13(6.5%)	38 (19.1%)	4 (2.0%)	42
Negative	3 (1.5%)	154 (77.4%)	6 (3.0%)	151 (75.9%)	157
Total	32	167	44	155	199

* Each indicates the percentile compared with the total 199 LNs.

#### Details of the discordance between the new and conventional systems and histopathology

Histopathology showed the following discordant diagnoses: 13 false-positive and 3 false-negative specimens (Table 4). In false-positive cases, the positive results of the 10 specimens were in agreement with those of the conventional OSNA system, which included small metastasis in isolated tumor cells (ITCs) and the allocation bias of cancer (ABC), where there were no tumor cells in the split surface used for pathological diagnosis. In the other three cases, contamination with epithelial tissue expressing CK19 mRNA occurred (C-22-1, B-14-3, and B-16-3). In false-negative cases, a metastasis of 2 mm was detected in the maximum diameter of surface B in one case (A-15-1); the presence of metastasis with necrosis led to RNA degradation in one case (A-12-1) and a strong inhibitory effect on the gene amplification reaction in one case (B-4-4).

**Table 4:** Discordance between OSNA and histology.

LN	OSNA		Histology				The reason of discordance
	New system	Conventional system	block B		block D		
			Judgement	metasitasis size	Judgement	metasitasis size	
A-9-2	+	+	-	ITC*	+	Macro^†^	ABC^§^
C-6-4	+	+	-	none	+	Macro	ABC^§^
C-16-3	+	+	-	none	+	Macro	ABC^§^
D-15-3	+	+	-	none	+	Macro	ABC^§^
B-2-1	+	+	-	ITC	-	none	ABC^§^
C-16-4	+	- →+^¶^	-	none	-	ITC	ABC^§^
A-12-3	+	+	-	none	-	none	ABC^§^
A-12-5	+	+	-	none	-	none	ABC^§^
A-15-4	+	+	-	none	-	none	ABC^§^
D-12-2	+	- →+	-	none	-	none	ABC^§^
C-22-1	+	+	-	none	-	none	CET^||^
B-14-3	+	+	-	none	-	none	CET
B-16-3	+	- →+	-	none	-	none	CET
A-15-1	-	-	+	Macro	-	none	small metastasis
A-12-1	-	-	+	Macro	n/a^‡^	n/a	RNA degradation
B-4-4	-	+	+	Macro	+	Macro	inhibition of gene amplification

* ITC; Isolated tumor cells,

†Macro; Macrometastasis,

‡n/a; The specimen peeled from the microscope slide,

§ABC; allocation bias of cancer,

|| CET; contamination with epithelial tissue,

¶ First and additional measurement

The concordance rate between the new and conventional systems was 95.0% with four different results (C-16-4, D-12-2, B-16-3, and B-4-4). In the additional measurement, the conventional system showed the same measurement in three specimens (C-16-4, D-12-2 and B-16-3), but one false-negative specimen (B-4-4) was correctly diagnosed as positive for metastasis using the conventional system.

### Influence of lung epithelial cells

By evaluating the pathological specimens of the lymph nodes, the contamination of lung epithelial cells was observed in the pathological preparation of 10 hilar lymph node specimens obtained from 9 patients with adenocarcinoma (Table 5).

**Table 5:** Lymph node specimens including lung epithelial cells.

LN	OSNA				Histology					
	New system		Conventional system		block B			block D		
	Judgement	CK19 mRNA (cCP/μl)	Judgement	CK19 mRNA (copy/μl)	Judgement	metasitasis size	contamination of lung epithelial (maximum diameter:mm)	Judgement	metasitasis size	contamination of lung epithelial (maximum diameter:mm)
C-16-5	+	2.5×10^5^	+	4.1×10^4^	+	Macro*	-	+	Macro	+ (10mm)
C-16-2	+	1.2×10^5^	+	3.0×10^4^	+	Macro	+ (10mm)	-	none	-
B-14-3	+	3.5×10^2^	+	1.0×10^3^	-	none	+ (3mm)	-	none	-
C-22-1	+	3.2×10^2^	+	6.9×10^2^	-	none	+ (1mm)	-	none	+ (4mm)
B-16-3	+	2.6×10^2^	-	<250	-	none	+ (4mm)	-	none	+ (3mm)
B-12-4	-	1.8×10^2^	+	3.2×10^2^	-	none	+ (2mm)	-	none	-
D-8-1	-	<160	+	<250	-	none	-	-	none	+ (2mm)
B-3-2	-	<160	-	<250	-	none	+ (9mm)	-	ITC^†^	-
C-13-1	-	<160	-	<250	-	none	+ (1mm)	-	none	-
B-9-2	-	<160	-	<250	-	none	+ (2mm)	-	none	-

*Macro; Macrometastasis,

†ITC; Isolated tumor cells

The specificities of eight negative lymph nodes, including the alveolar epithelium, were 62.5% (5/8) in the new system and 50.0% (4/8) in the conventional system, which were similar.

### Analysis of small lymph nodes

Among 186 specimens, measuring the short diameter at the time of lymph node dissection, we found that the larger the diameter, the higher the rate of positive metastasis (S1 Fig). Among the 94 lymph nodes with a diameter of less than 10 mm, eight specimens were judged as positive by histopathology and six were judged as positive by the new system. Among the two specimens judged as positive by histopathology and negative by the new system, one had cancer observed only on surface B, while the other had only necrosis leading to RNA degradation. Among the six specimens judged as negative by histopathology and positive by the new system, two had micrometastasis (ITC), which was judged as positive by the conventional system (Table 6).

**Table 6:** Ninety-four lymph nodes with a diameter less than 10 mm that were judged as positive by histology or the OSNA new system.

Histology using two surfaces		OSNA new system		The reason of discordance	
Positive	8	Positive	6		
		Negative	2	small metastasis, RNA degradation	
Negative	86	Positive	6	ITC* in 2, ABC^†^ in 4	
		Negative	80		

* Isolated tumor cells,

†allocation bias of cancer

## Discussion

OSNA is a useful diagnostic procedure for the detection of lymph node metastasis. OSNA using RD-100i/LYNOAMP BC has shown clinical performance equivalent to that of histopathology in the examination of regional lymph node metastasis in patients with NSCLC [5–7]; the concordance rate between two method was 92.7% [7]. The new system, RD-200/LYNOAMP CK19, showed the same performance capabilities in diagnosing breast, gastric, and colon cancer. In this study, for the first time, we evaluated the clinical performance of the new OSNA system at multiple centers because no study has verified the clinical performance of this new system in the diagnosis of lymph node metastasis of NSCLC. The new system was compared with histopathology, and the concordance rate was 92.0%, which was equivalent to that of the conventional system; the allocation bias of cancer in each lymph node is a discordance unique to this study due to the experimental method of dividing the lymph nodes into OSNA and histopathology and does not occur when the entire lymph node is measured in clinical practice. Although histopathology with a limited surface may overlook the presence of metastasis because of the allocation bias of the cancer and underestimate the postoperative staging, OSNA can evaluate the entire lymph node without the influence of allocation bias and increase the detection rate of metastasis-positive lymph nodes.

For the 10 false-positive specimens, contamination of the alveolar epithelium and metastasis to other lymph nodes were not observed; although the conventional system showed a positive result consistent with that of the new system, there is a possibility that micrometastasis was localized only in the blocks used for OSNA.

However, contamination of the alveolar epithelium was observed as a factor of discordance with histopathology, evaluated in this study. Eight histopathologically negative specimens included the alveolar epithelium, three of which showed false-positive results using the new system. The specificities for negative lymph nodes, including the alveolar epithelium, were similar between the new and conventional systems. Since the effect on the OSNA assay depends on the level of CK19 mRNA expression in the lymph nodes and in the alveolar epithelium and the amount of alveolar epithelium, contamination of the alveolar epithelium does not necessarily indicate a false-positive result; however, the alveolar epithelium should be removed by carefully trimming the area along the coat to prevent false-positive results.

Three false-negative events (A-15-1, A12-1, and B-4-4) were unique to each lymph node. Since these three metastases were small but not ITC, it is considered there is no correlation between the size of the metastases and CK19 levels. The results were probably caused by RNA degradation due to necrosis, and inhibition of gene amplification by substances in the lymph node lysate. Lymph nodes in which necrosis is strongly suspected after resection should not be used in the OSNA assay, and the assay should be performed immediately after lymph node resection to prevent the degradation of RNA quality. If it is difficult to examine immediately, the resected specimen should be stored in a cold environment. For the specimen with a false-negative result (B-4-4), CK19 mRNA was detected within the measurement time in both systems, while the inhibition of the gene amplification reaction by the components in the lymph node lysate was different in both systems; the new system showed a false-negative result but was eventually converted to a positive result using a diluted specimen. There were some factors that inhibited the gene amplification reaction in this specimen, and the factors were also diluted together. However, we could not identify the factor this time; it is an extremely rare event, but it is possible. This tumor had low expression of CK-19 and wide necrosis, which led to a false-negative result, and it can be detected using the conventional system even with multiple measurements. Detection of a tumor that does not show gene amplification reaction was difficult, but caution should be exercised if a tumor has strong necrosis or low CK-19 expression.

The judgment of three specimens (C-16-4, D-12-2, and B-16-3) changed from the first assessment to the multiple assessments using the conventional system, but it was the same in the new system. The new system seemed to be better than the conventional system in terms of reproducibility near the cut-off with low-copy samples, including the foreign element. However, the diagnostic outcomes of the conventional and new systems were equivalent in this study, the high reproducibility of the new system will lead to higher reliability of each examination.

There was a correlation between the short diameter of the lymph node and the positive rate, but positive lymph nodes were found even when the diameter was less than 10 mm, which suggests that the accuracy of the presence or absence of metastasis cannot be ensured only by estimating the short diameter of the lymph node as a preoperative diagnosis. Because the probability of obtaining false-negative results is low compared with the short diameter of the lymph node and the new system has a good concordance rate of 94.7% (89/94) compared with the histopathological results of the lymph node specimens with a diameter of less than 10 mm, lymph node metastasis can be more accurately detected using the new system than histology. The OSNA method, which uses the whole lymph node to examine, can detect micrometastasis even in the small lymph node, but more attention should be paid when removing pulmonary substances.

The high sensitivity of the OSNA assay in the postoperative staging of colorectal cancer is also useful for predicting the risk of recurrence in patients with pStage II [15]. The OSNA assay is globally used in patients with breast cancer not only to detect lymph node metastasis in order to determine whether axillary dissection should be performed but also to predict axillary lymph node metastasis and prognosis from the total tumor load of the total CK19 mRNA expression in a few sentinel lymph nodes [16–25]. These findings suggest that CK19 mRNA expression not only indicates lymph node metastasis but also provides new values, such as the degree of cancer progression, which is not detected in histopathological analyses. Based on the high NPV of the OSNA assay, patients with N0 assessed using OSNA have better prognosis than those with N0 assessed using histopathology [25]. Since the prognosis of lung cancer is associated with lymph node metastasis, the results obtained by the OSNA assay can be used to predict the prognosis of lung cancer.

Overall, we believe that intraoperative diagnosis can best demonstrate the benefits of OSNA. The results of JCOG0802/WJOG4607L, a multicenter randomized controlled trial, revealed that OS in the segmentectomy group was significantly better than that in the lobectomy group for lung cancers smaller than 2 cm; the results were first presented at the American Association for Thoracic Surgery meeting in 2021. In the next year, the results of a similar CALGB140305 trial in North America will be reported. Although it will depend on the results of these trials, the importance of segmentectomy in small lung cancers is likely to increase. It is necessary to prove that there are no metastases in the hilar lymph nodes because lobectomy remains the standard procedure with N1 status. Since it is difficult to evaluate small lymph nodes using positron emission tomography, intraoperative evaluation is required. OSNA can used to diagnose lymph node metastasis with small lesions or location bias, even in facilities where intraoperative rapid tests are not available.

Diverse histology is a feature of lung cancer and there may be variation in CK19 expression. Nakagawa et al. reported that the clinical performance of the OSNA assay for rare cancer types other than adenocarcinoma and squamous cell carcinoma requires additional evaluation owing to the limited number of low-incidence histological types because there were some cases with low CK19 expression in pleomorphic carcinoma and large cell carcinoma [7]. Despite the limited number of 16 cases, CK19 expression in primary tumors was found to be highly in all (Supl.2). Two cases of pleomorphic carcinoma and large cell carcinoma, which were diagnosed NSCLC preoperatively, had high CK19 expression in this study. For such rare histological types, there may be differences in CK19 expression between cases [6, 7] The indications for rare histological types will have to be evaluated in more cases continuously. If preoperatively diagnosed with NSCLC other than adenocarcinoma or squamous cell carcinoma, it may be better to check for CK19 expression with immunostaining examination. If the expression can be confirmed, the reliability of OSNA will be improved.

As a limitation of this study, the lymph nodes were collected from surgical patients with stage II or lower lymph node metastasis and few metastasis-positive specimens. Thus, further studies and analyses using more cases are needed, including many histological types.

## Conclusions

This is the first study to evaluate the performance of the new OSNA system in patients with NSCLC. Since this system can examine the lymph nodes more and faster than the conventional system, and its performance is comparable with to that of histopathology and the conventional system, it can be effectively utilized during lung cancer treatment intraoperatively and postoperatively. It is considered this new OSNA system could be the gold standard technique for the diagnosis of lymph node metastasis.

## Supporting information

S1 Fig Comparison between lymph node size and positive rate assessed using histology. (JPG)

S1 Table Cytokeratin19 expression in primary tumor among 16 cases. (DOCX)

## List of abbreviations

ABC: allocation bias of cancer
CI: confidence interval
CK19: cytokeratin 19
CT: computed tomography
ITC: isolated tumor cells
NSCLC: non-small cell lung cancer
NPV: negative predictive value
LN: lymph node
PPV: positive predictive value
OSNA: one-step nucleic acid amplification
