# Supplementary figures and images for: Detection of lymph node metastasis in non-small cell lung cancer using the new system of one-step nucleic acid amplification assay
Source: PLoS One. 2022 Mar 21;17(3):e0265603. doi: 10.1371/journal.pone.0265603 (PMC8936453; doi:10.1371/journal.pone.0265603)

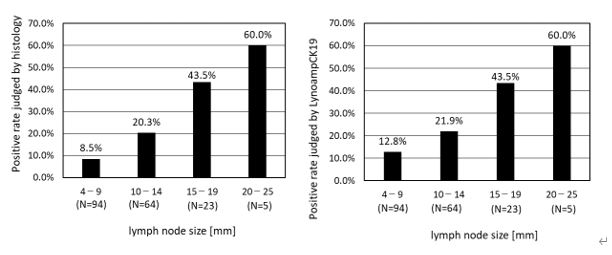

Supplement: S1 Fig — (JPG) [file pone.0265603.s001.JPG]
