# Supplementary material for: Detection of lymph node metastasis in non-small cell lung cancer using the new system of one-step nucleic acid amplification assay
Source: PLoS One. 2022 Mar 21;17(3):e0265603. doi: 10.1371/journal.pone.0265603 (PMC8936453; doi:10.1371/journal.pone.0265603)
Supplement: S1 Table — (DOCX) [file pone.0265603.s002.docx]

S1 Table. Cytokeratin19 expression in primary tumor among 16 cases

| sample | histogical type | equivalent to  cut-off value* | | CK19mRNA /1.5mm square (cCP/ul) |
| --- | --- | --- | --- | --- |
|  |  | tissue volume(mg) | mm square |  |
| 1 | Sq | 0.04 | 0.34 | 58300 |
| 2 | Pleo | 2.51 | 1.36 | 3060 |
| 3 | Ad | 3.66 | 1.51 | 2360 |
| 4 | Ad | 0.88 | 0.96 | 10300 |
| 5 | Sq | 0.52 | 0.80 | 13600 |
| 6 | Ad | 1.31 | 1.10 | 5800 |
| 7 | Ad | 0.03 | 0.29 | 91700 |
| 8 | Sq | 0.21 | 0.59 | 55600 |
| 9 | Sq | 0.25 | 0.63 | 19700 |
| 10 | Ad | 3.82 | 1.56 | 2250 |
| 11 | Sq | 0.91 | 0.97 | 8330 |
| 12 | Sq | 2.96 | 1.44 | 2555 |
| 13 | Sq | 9.00 | 2.08 | 917 |
| 14 | Ad | 3.66 | 1.54 | 2250 |
| 15 | Large | 0.20 | 0.58 | 23900 |
| 16 | Sq | 0.12 | 0.49 | 18900 |
| Ad; adenocarcinoma, Sq; squamous cell carcinoma,  Pleo; pleomorhic carcinoma, Large; large cell carcinoma *the cut-off value is 250cCP/ul. | | | | |
